# Holistic Human-Serving Digitization of Health Care Needs Integrated Automated System-Level Assessment Tools

**DOI:** 10.2196/50158

**Published:** 2023-12-20

**Authors:** Cindy Welzel, Fabienne Cotte, Magdalena Wekenborg, Baptiste Vasey, Peter McCulloch, Stephen Gilbert

**Affiliations:** 1 Else Kröner Fresenius Center for Digital Health TUD Dresden University of Technology Dresden Germany; 2 Ada Health GmbH Berlin Germany; 3 Nuffield Department of Surgical Sciences University of Oxford Oxford United Kingdom

**Keywords:** health technology assessment, human factors, postmarket surveillance, software as a medical device, digital health tools, quality assessment, quality improvement, regulatory framework, user experience, health care

## Abstract

Digital health tools, platforms, and artificial intelligence– or machine learning–based clinical decision support systems are increasingly part of health delivery approaches, with an ever-greater degree of system interaction. Critical to the successful deployment of these tools is their functional integration into existing clinical routines and workflows. This depends on system interoperability and on intuitive and safe user interface design. The importance of minimizing emergent workflow stress through human factors research and purposeful design for integration cannot be overstated. Usability of tools in practice is as important as algorithm quality. Regulatory and health technology assessment frameworks recognize the importance of these factors to a certain extent, but their focus remains mainly on the individual product rather than on emergent system and workflow effects. The measurement of performance and user experience has so far been performed in ad hoc, nonstandardized ways by individual actors using their own evaluation approaches. We propose that a standard framework for system-level and holistic evaluation could be built into interacting digital systems to enable systematic and standardized system-wide, multiproduct, postmarket surveillance and technology assessment. Such a system could be made available to developers through regulatory or assessment bodies as an application programming interface and could be a requirement for digital tool certification, just as interoperability is. This would enable health systems and tool developers to collect system-level data directly from real device use cases, enabling the controlled and safe delivery of systematic quality assessment or improvement studies suitable for the complexity and interconnectedness of clinical workflows using developing digital health technologies.

## Introduction

Digital health tools (DHTs) and software as a medical device (SaMD), including artificial intelligence (AI)–enabled medical devices (AIeMDs), have great potential to improve health care. These tools, however, have often been designed with limited interoperability and limited optimization to location-specific clinical workflows and approaches [1]. Although this also applies to some physical medical devices, for which the product-focused regulatory and health technology assessment (HTA) frameworks were first developed, it particularly applies to SaMD [2].

Physical medical devices are generally standalone tools, specifically designed systems, or collated procedure packs of devices for a specific purpose. They do not have a natural need for general interoperability in the same manner that digital systems do, where a congruent flow of data through systems is needed to avoid reentry and error [3]. Software systems require a system view evaluation, as recognized in the call for the regulation of AIeMDs [4]. This need has also been recognized from the HTA perspective, where there have been calls for a more holistic “total product lifecycle approach” [5], emphasizing the consideration of the entire lifecycle of tools from premarket development via postmarket surveillance (PMS) of real-use contexts to disinvestment. Collaboration and patient involvement are key factors in this approach, which requires systematic evaluation of the value and effectiveness of clinical benefits, risks, and costs at each stage, alongside assessment of the impact on quality of care and health care resource use [5]. The literature on regulation and HTA recognizes the importance of a standardized and holistic “system view,” which is partially reflected in frameworks [6,7] as well as in the approaches of HTA and PMS. However, the practical application of this holistic “system view” has been limited so far. This approach will require holistic system-level and location-specific analyses of diverse real-use scenarios of interacting digital tools in health care.

The UK National Health Service has introduced digital technology assessment criteria for health and social care to ensure clinical safety, and their framework specifically recognizes interoperability as well as usability and accessibility of DHTs [8]. The framework is applied at the time of procurement rather than for continuous assessment, and neither tool developers, regulators, nor HTA agencies currently have incentives or resources to carry out whole system–level analyses. Health systems apply system-level quality assessment (QA) and quality improvement (QI) exercises in a patchwork fashion, unlinked to either the regulatory approval of the digital tools or their HTA or reimbursement. Proposed US legislation would require larger health systems, along with developers, to holistically and systematically assess AIeMDs and algorithm-based automated systems in real-world use cases, considering interoperability [9].

We propose that such models should be linked to regulatory approval status and HTA for DHTs. Moreover, we propose that standardized approaches for this system-level assessment could be built into the assessed tools themselves through requirements for interoperability and data standards that already exist in some countries [3]. We set out here a model of a standardized system-level assessment approach and show how this could be used to automate health system QA and improvement studies. As health care is becoming increasingly automated through digital systems, the degree to which these systems work for patients and providers should also be measurable “at the touch of a button” through automated digital assessment systems.

## What Happens When DHTs Are Badly Designed for Their Human Users?

Health care providers (HCPs) are among the occupational groups most strongly affected by chronic work stress and its associated pathologies [10], posing a severe threat to their ability to work and thus to the functioning of the entire health care system. DHTs have the potential to reduce this stress by making certain tasks and responsibilities less burdensome [11]. However, they could also prove to do the contrary if not properly designed and evaluated. The performance of DHTs and AIeMDs (eg, clinical decision support systems) is not only dependent on the underlying software algorithm but also on how systems interact with and are operated by the users [12]. These human factors (HF) influence usability and include psychological, cognitive, and social factors [12]. They determine the relationship between humans and the tools they use. HF research and optimization aims for a better understanding and design of the interaction between health care professionals and the tools they use at the cognitive, social, and organizational levels [13].

HF research on the social level includes the interaction of people in a specific setting. For example, in health care, patients interact with physicians, psychotherapists, physiotherapists, and nurses. The communicative and trustful interaction of these actors assists in the delivery of optimal care [14-16]. HF at the cognitive level comprises the users’ perception of the DHT including in terms of its design and usability. For example, HF includes the exploration of whether the user interface design is intuitive and easy to use or if it is too complex and therefore poses a high risk of operating errors, leading to patient harm [14-16]. Indeed, the success or failure of DHTs is largely predicated on the end user acceptability of the introduced technologies, which reinforces the importance of HF research [12].

**Figure 1:**
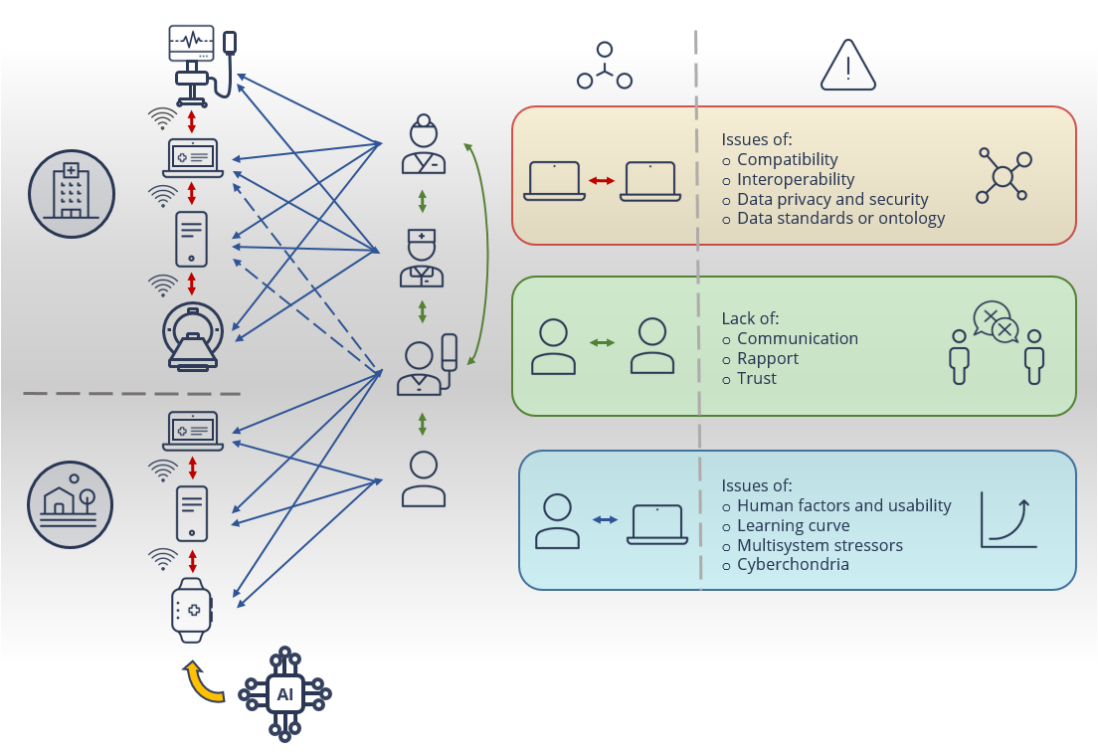
Interaction network of digital health tools with their different users (doctors, nurses, patients, and citizens) and potential "technostressors." "Cyberchondria" refers to a clinical phenomenon where repeated internet searches regarding medical information result in excessive concerns about physical health [22].

Preliminary findings indicate that the implementation of DHTs often has a stress-enhancing effect [17,18]. They can result in so-called technostress in HCPs (ie, the inability to cope with the requirements of digital technology) [19]. A contributory factor to HCP technostress is poor interoperability between DHTs, that is, the ability of 2 or more tools to exchange and use information [20,21]. Particularly relevant to HCP stress is semantic interoperability, meaning the exchange and use of information with consistent and uniform meaning [21]. DHTs are increasingly part of health delivery approaches, and these tools are not only deeply embedded in clinic-specific workflows but also frequently used by patients and citizens from home as wellness apps (eg, fitness or nutrition apps). These tools interact with each other as well as with their users (Figure 1). The manner in which they integrate into existing or adapted clinical routines is crucial to avoid interoperability, communication, and usability issues (Figure 1). This depends on both technical and HF aspects, and therefore it is important to provide intuitive and safe user interface designs for DHTs and to ensure that these tools are usable in practice.

## Postmarket HF Assessment Particularly Important for On-Market Adaptive Tools

Software is changed and adapted more over time than hardware-based medical devices, and AIeMDs are particularly subject to change, as they are based on prediction models that improve through retraining on new data. This adaptation based on feedback or data is an advantage but is also challenging in a medical setting, where proven performance based on clinical outcomes and safety data is required. The quality of output and accuracy of many DHTs are highly dependent on the correct use of the device. SaMD developers must carry out extensive HF research to minimize the amount of training or help required when using DHTs [23,24]. Once the tool is on the market, developers typically conduct internal automated testing before releasing new versions. Such testing can provide data on the accuracy of AI prediction models in isolation but can only evaluate the human-AI team to a limited degree, and HF is a critical missing link between computational performance and clinical outcomes [25,26]. Developers rarely repeat comprehensive HF assessments with real users, such as patients and health care professionals for minor changes. Over time, minor changes cumulatively become major changes, which could lead to reduced efficacy and open up unforeseen risks.

## Existing Strategies for HTA and Real-World Performance Monitoring

Active PMS approaches enable the monitoring of the real-world performance of DHTs in their real-use environment, theoretically including system-level interactions. Currently, this is generally through ad hoc approaches like investigator-initiated studies and other forms of clinical investigations. These often use validated surveys completed by patients and HCPs that can be implemented directly in the DHT, allowing seamless data collection as these are often networked devices with a user interface (UI) [27]. Validated survey instruments, including patient-reported outcome measures (PROMs) and patient-reported experience measures (PREMs), as well as clinician-reported outcomes (CROs) and clinician-reported experience measures (CREMs) serve as standardized, questionnaire-based self-reporting instruments [27]. The data generated by these measures are used by clinicians and other health care administrators to evaluate the effectiveness, appropriateness, and acceptability of the investigated therapy and identify areas for QI. These approaches can be implemented as digital surveys in digital devices and can be used to collect data on their safety, performance, cost-effectiveness, efficiency, and usability.

Although these ad hoc approaches are valuable in providing evidence and addressing patient perceptions and HCP stress, each DHT developer focuses on their own issues and develops their own evaluation approaches. This is inefficient and generates data silos that exist across the health evidence ecosystem [28], which results in multiple generations of overlapping evidence without interlinking this evidence across the systems or to other DHT developers, and often these data are not shared with the health systems. This creates challenges in efficient data sharing and results in communication barriers [29]. Existing data silos often result in researchers generating evidence for questions that are already answered or that are not priorities for decision-makers [29]. System-level data collection approaches are needed to enable efficient, systematic, and standardized postmarket collection of data on the real-world performance of DHTs in diverse health systems.

## Approach for Automated and Standardized System-Level Assessment

The problems we have outlined will be challenging to address without cost-efficient automated systems for cross-system data collection. Such systems are unlikely to be created by individual DHT developers but could be required by HTA and regulatory bodies. Common integrated data collection systems built on standard platforms would enable a higher level of collaboration between HTA and regulatory bodies, payers, and other health care system stakeholders and help ensure that data and findings are accessible in an efficient and transparent manner and that flexible and adaptive response to new evidence is possible (Textbox 1).

**Requirements for a system-level postmarket surveillance and health technology assessment approach [5].**

- Collaboration within and between regulatory and postmarket surveillance bodies and the wider health care system involving all stakeholders
  (eg, clinicians, caregivers, patients, and society)
- Standardization of evidence requirements and frameworks and development of universal core approaches for all technologies
- Transparency in postmarket surveillance and health technology assessment policies, procedures, and outputs to allow data sharing within and across jurisdictions
- Infrastructure for efficient use and sharing of data, including real-world data and real-world evidence

**Figure 2:**
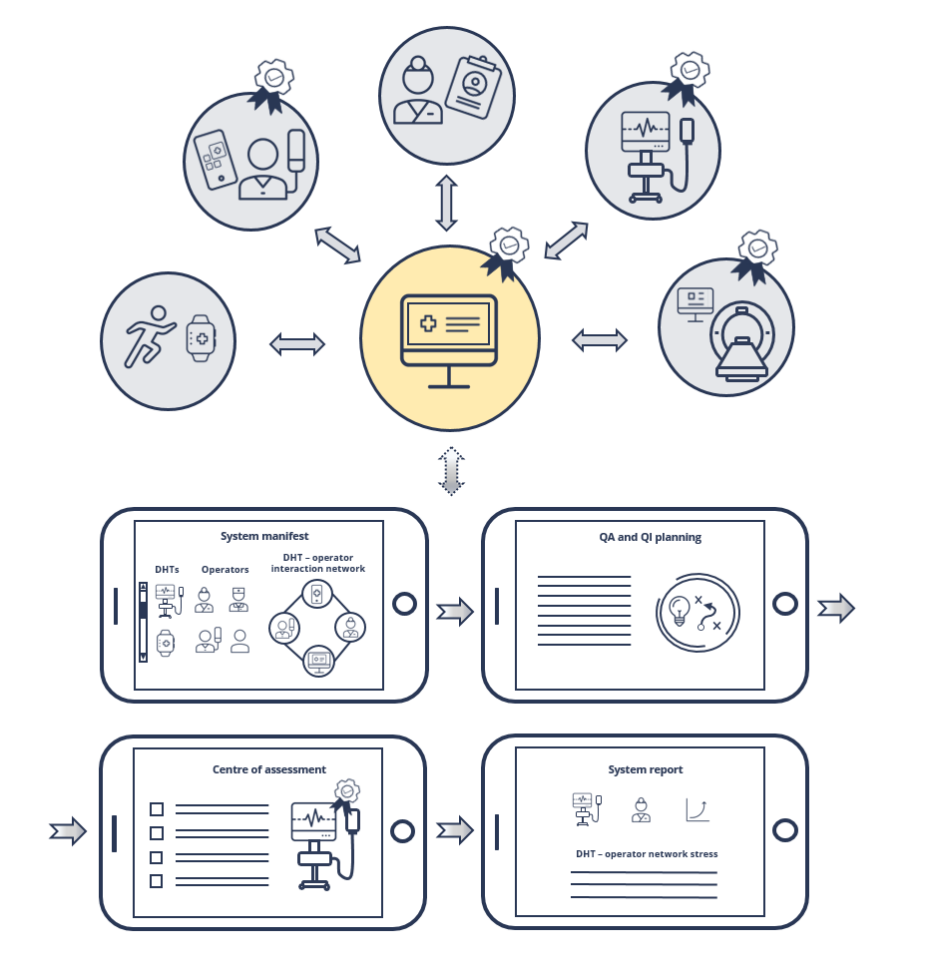
Approach for a system-level postmarket surveillance and health technology assessment QA or QI framework. The first screen view shows the users of the DHT and how they interact; the second screen view shows the QA or QI planning phase; the third screen view shows questionnaire-based QA, including patient-reported outcome measures, patient-reported experience measures, clinician-reported experience measures, and clinician-reported outcomes; the fourth screen view shows a system report with stressors that affect the user-DHT interaction and need to be improved. DHT: digital health tool; QA: quality assessment; QI: quality improvement.

A common and international system could be developed by a combination of regulatory and HTA bodies, which could enable the delivery of validated instruments for measuring PROMs, PREMs, CREMs, and CROs in a coordinated fashion (Figure 2). This system could be made available through regulatory and HTA bodies as an application programming interface to developers and could be stipulated as a requirement for DHTs, similarly to the requirement for including standard approaches for interoperability and interfacing.

The proposed system would provide standardized e-questionnaires with the ability for stakeholders (health care systems, regulatory and HTA bodies, and developers) to efficiently build cross-system integrated questionnaires, delivering PROM, PREM, CREM, and CRO measures particularly in the case of the assessment of HCP-facing DHTs but also for patient-facing apps in a clinical context. They could either appear as pop-ups directly on the device UI in the electronic health record (EHR) or as a separate questionnaire delivered through a context-specific QI study coordination web interface (Figure 2). Relevant HFs, like user stress and interoperability, could be assessed through these questionnaires, automated use reports, and performance metrics, with data collected across multiple system manufacturers and clinical interfaces.

The interlinking of assessment based on a manifest of all interlinked DHTs in use in the clinical center (by HCPs and patients) would allow the holistic collection and assessment of outcome and experience measures and better take into account the complexity of stress experiences of patients and HCPs. Additionally, the assessment data could, where relevant, be transmitted to the respective regulatory and HTA bodies as well as to public health organizations like the National Centers for Disease Control and Prevention for secondary use of health data. This can improve the overall standard of health care by enhancing health care experiences for patients, expanding knowledge about diseases and appropriate treatments, strengthening the understanding of effectiveness and efficiency of health care systems, supporting public health and security goals, and aiding businesses in meeting customers’ needs.

The holistic system we propose is intended not only for the assessment of different kinds of HCP-facing DHTs like clinical decision support systems but also EHRs and patient portals, which may also contain patient-facing elements. Although basic principles for HF, usability, and interoperability assessment remain the same, different technologies and applications of DHTs necessitate the adaptation of the evaluation process, which could become complex given the growing spectrum of DHTs. To realize this, a toolbox could be implemented, offering a set of standardized tools like questionnaires using PROMs, PREMs, CROs, and CREMs. Regulatory and HTA bodies or developers could choose appropriate tools for the respective DHT. The selected set of tools could then be delivered to the HCP through an application programming interface and presented as a pop-up directly on the device UI in the EHR or as a separate questionnaire delivered through a web interface. AIeMDs represent a special case because adaptation based on learning is a basic principle of this technology. To address the role of adaptability in AI technologies, a continuous assessment is necessary, which could be realized by the implementation of predetermined change control plans into the proposed holistic system [2,30-32].

## Safe Automated Assessment Delivery and the Human Role

Clearly, the delivery of validated survey instruments through the UI of DHTs, some of which are safety-critical tools, requires care and forethought; otherwise, the automated digital QA or QI approach would be an additional and large stressor for HCPs and a safety concern. All users of general apps and websites are aware of the irritation that can be caused by recurrent pop-up feedback surveys. In DHTs, approaches have been developed for the safe delivery of (non–network coordinated) surveys. We anticipate that our proposed approach for automated and standardized system-level assessment would be used as part of human-planned, preannounced, and efficient-to-deliver QA or QI exercises, which would include automated and human-verified safety and burden controls. The described PMS and HTA system must ensure user privacy, especially for patients, and be implemented in a General Data Protection Regulation–compliant manner. Since the system is not intended for individually assessing the performance of HCPs, the information gathered from questionnaires would be grouped and presented in a way that avoids revealing specific HCP identities. Likewise, the findings linked to patient information could be aggregated. In case there is a need to thoroughly examine significant safety issues concerning particular patients, established General Data Protection Regulation–compliant procedures for root cause analysis would be followed.

## Technical Implementation

The described holistic PMS and HTA approach is fully technically achievable. For data use and storage, existing secure and trusted technological identity management approaches are applicable. Secure cloud-based interfaces such as those already commonly used to manage credit card transactions can link out to external apps or overlay browser screens, with highly automated 1-time password systems for authentication and security [33]. These systems enable external apps to interact with the DHTs in a highly secure manner via standardized authentication protocols (including hooks). This allows the secure execution of predetermined tasks on DHTs and allows the predetermined secure sharing of structured data between DHTs. The holistic PMS and HTA evaluation approach proposed would also enable interoperability and interaction with legacy and nondigital systems through standardized questionnaires delivered via a common QA or QI web interface on HCP desktop or tablet computers.

## Summary

By combining subjective user feedback with objective data from the DHTs, a more complete and holistic view of the performance of a health care technology could be obtained. This would enable health systems and tool developers to collect system-level data through an automated assessment system linked to real device use cases. This approach would also allow the controlled and safe digital delivery of systematic QA or QI studies suitable for assessing complex clinical workflows and the nature of human interactions with the increasingly interconnected network of HCP- and patient-facing DHTs. Any system-level digital approach to assessment is likely to face pushback and criticism that it is expensive to develop or time consuming to operate. In recent years, system-level requirements for interoperability of DHTs have been introduced in some countries [34], and the European Health Data Space will introduce very substantial requirements for data structuring, interoperability, and sharing [35]. For a less fragmented and more fit-for-purpose approach for monitoring the multideveloper ecosystem of modern digital health delivery to emerge, courage and investment are needed to develop shared and networked system-level assessment of interacting DHTs.

## Abbreviations

AI: artificial intelligence
AIeMD: AI-enabled medical device
CRO: clinician-reported outcome
CREM: clinician-reported experience measure
DHT: digital health tool
EHR: electronic health record
HCP: health care provider
HF: human factors
HTA: health technology assessment
PMS: postmarket surveillance
PROM: patient-reported outcome measure
PREM: patient-reported experience measure
QA: quality assessment
QI: quality improvement
SaMD: software as a medical device
UI: user interface

## References

[1] Vasey B, Ursprung S, Beddoe B, Taylor EH, Marlow N, Bilbro N, Watkinson P, McCulloch P (2021). Association of clinician diagnostic performance with machine learning-based decision support systems: a systematic review. JAMA Netw Open.

[2] Gilbert S, Fenech M, Hirsch M, Upadhyay S, Biasiucci A, Starlinger J (2021). Algorithm change protocols in the regulation of adaptive machine learning-based medical devices. J Med Internet Res.

[3] Interoperability standards in digital health—a white paper from the medical technology industry. MedTech Europe.

[4] Gerke S, Babic B, Evgeniou T, Cohen IG (2020). The need for a system view to regulate artificial intelligence/machine learning-based software as medical device. NPJ Digit Med.

[5] Trowman R, Migliore A, Ollendorf DA (2023). Health technology assessment 2025 and beyond: lifecycle approaches to promote engagement and efficiency in health technology assessment. Int J Technol Assess Health Care.

[6] The MDR's usability/human factors requirements. Johner Institute.

[7] (2019). Applying human factors and usability engineering to medical devices. U.S. Food and Drug Administration.

[8] Digital Technology Assessment Criteria. NHS Transformation Directorate.

[9] (2023). Marketing submission recommendations for a predetermined change control plan for artificial intelligence/machine learning (AI/ML)-enabled device software functions. U.S. Food and Drug Administration.

[10] Rotenstein LS, Torre M, Ramos MA, Rosales RC, Guille C, Sen S, Mata DA (2018). Prevalence of burnout among physicians: a systematic review. JAMA.

[11] McKee M, van Schalkwyk MCI, Stuckler D (2019). The second information revolution: digitalization brings opportunities and concerns for public health. Eur J Public Health.

[12] Kushniruk AW, Borycki EM (2023). Human factors in healthcare IT: management considerations and trends. Healthc Manage Forum.

[13] Schueller SM (2021). Grand challenges in human factors and digital health. Front Digit Health.

[14] Scheder-Bieschin J, Blümke B, de Buijzer E, Cotte F, Echterdiek F, Nacsa J, Ondresik M, Ott M, Paul G, Schilling T, Schmitt A, Wicks P, Gilbert S (2022). Improving emergency department patient-physician conversation through an artificial intelligence symptom-taking tool: mixed methods pilot observational study. JMIR Form Res.

[15] Montazeri M, Multmeier J, Novorol C, Upadhyay S, Wicks P, Gilbert S (2021). Optimization of patient flow in urgent care centers using a digital tool for recording patient symptoms and history: simulation study. JMIR Form Res.

[16] Cotte F, Mueller T, Gilbert S, Blümke B, Multmeier J, Hirsch MC, Wicks P, Wolanski J, Tutschkow D, Brittinger CS, Timmermann L, Jerrentrup A (2022). Safety of triage self-assessment using a symptom assessment app for walk-in patients in the emergency care setting: observational prospective cross-sectional study. JMIR Mhealth Uhealth.

[17] Babbott S, Manwell LB, Brown R, Montague E, Williams E, Schwartz M, Hess E, Linzer M (2014). Electronic medical records and physician stress in primary care: results from the MEMO study. J Am Med Inform Assoc.

[18] Heponiemi T, Hyppönen H, Kujala S, Aalto AM, Vehko T, Vänskä J, Elovainio M (2018). Predictors of physicians' stress related to information systems: a nine-year follow-up survey study. BMC Health Serv Res.

[19] Brod C (1984). Technostress: The Human Cost of the Computer Revolution.

[20] (1991). IEEE Standard Computer Dictionary: A Compilation of IEEE Standard Computer Glossaries, 610.

[21] Lehne M, Sass J, Essenwanger A, Schepers J, Thun S (2019). Why digital medicine depends on interoperability. NPJ Digit Med.

[22] Mathes BM, Norr AM, Allan NP, Albanese BJ, Schmidt NB (2018). Cyberchondria: overlap with health anxiety and unique relations with impairment, quality of life, and service utilization. Psychiatry Res.

[23] Formicola R, Amici C, Mor M, Bissolotti L, Borboni A (2023). Design of medical devices with usability in mind: a theoretical proposal and experimental case study using the LEPRE device. Designs.

[24] Shin J, Lee H (2023). Optimal usability test procedure generation for medical devices. Healthcare (Basel).

[25] Berkovsky S, Coiera E (2023). Moving beyond algorithmic accuracy to improving user interaction with clinical AI. PLOS Digit Health.

[26] Vasey B, Nagendran M, Campbell B, Clifton DA, Collins GS, Denaxas S, Denniston AK, Faes L, Geerts B, Ibrahim M, Liu X, Mateen BA, Mathur P, McCradden MD, Morgan L, Ordish J, Rogers C, Saria S, Ting DSW, Watkinson P, Weber W, Wheatstone P, McCulloch P (2022). Reporting guideline for the early-stage clinical evaluation of decision support systems driven by artificial intelligence: DECIDE-AI. Nat Med.

[27] Lewis A, Valla V, Charitou P, Karapatsia A, Koukoura A, Tzelepi K, Bergsteinsson JI, Ouzounelli M, Vassiliadis E (2022). Digital health technologies for medical devices—real world evidence collection—challenges and solutions towards clinical evidence. Int J Digit Health.

[28] Bevc CA, Retrum JH, Varda DM (2015). New perspectives on the "silo effect": initial comparisons of network structures across public health collaboratives. Am J Public Health.

[29] Vatanpour H, Khorramnia A, Forutan N (2013). Silo effect a prominence factor to decrease efficiency of pharmaceutical industry. Iran J Pharm Res.

[30] Gilbert S, Pimenta A, Stratton-Powell A, Welzel C, Melvin T (2023). Continuous improvement of digital health applications linked to real-world performance monitoring: safe moving targets?. Mayo Clinic Proc Digit Health.

[31] Gilbert S, Anderson S, Daumer M, Li P, Melvin T, Williams R (2023). Learning from experience and finding the right balance in the governance of artificial intelligence and digital health technologies. J Med Internet Res.

[32] Wall DP, Liu-Mayo S, Salomon C, Shannon J, Taraman S (2023). Optimizing a de novo artificial intelligence-based medical device under a predetermined change control plan: improved ability to detect or rule out pediatric autism. Intell Based Med.

[33] Paterson KG, Stebila D, Steinfeld R, Hawkes P (2010). One-time-password-authenticated key exchange. Information Security and Privacy. ACISP 2010. Lecture Notes in Computer Science, vol 6168.

[34] FHIR, phase I—DiGA toolkit 1.0.0. MIO.

[35] (2022). Proposal for a regulation of the European parliament and of the council on the European health data space. EUR-Lex.

